# Supplementary material for: In Silico Screening of Natural Flavonoids against 3-Chymotrypsin-like Protease of SARS-CoV-2 Using Machine Learning and Molecular Modeling
Source: Molecules. 2023 Dec 10;28(24):8034. doi: 10.3390/molecules28248034 (PMC10745665; doi:10.3390/molecules28248034)
Supplement: Supplementary file 1 [file molecules-28-08034-s001.zip › Figures S1-S9.pdf]

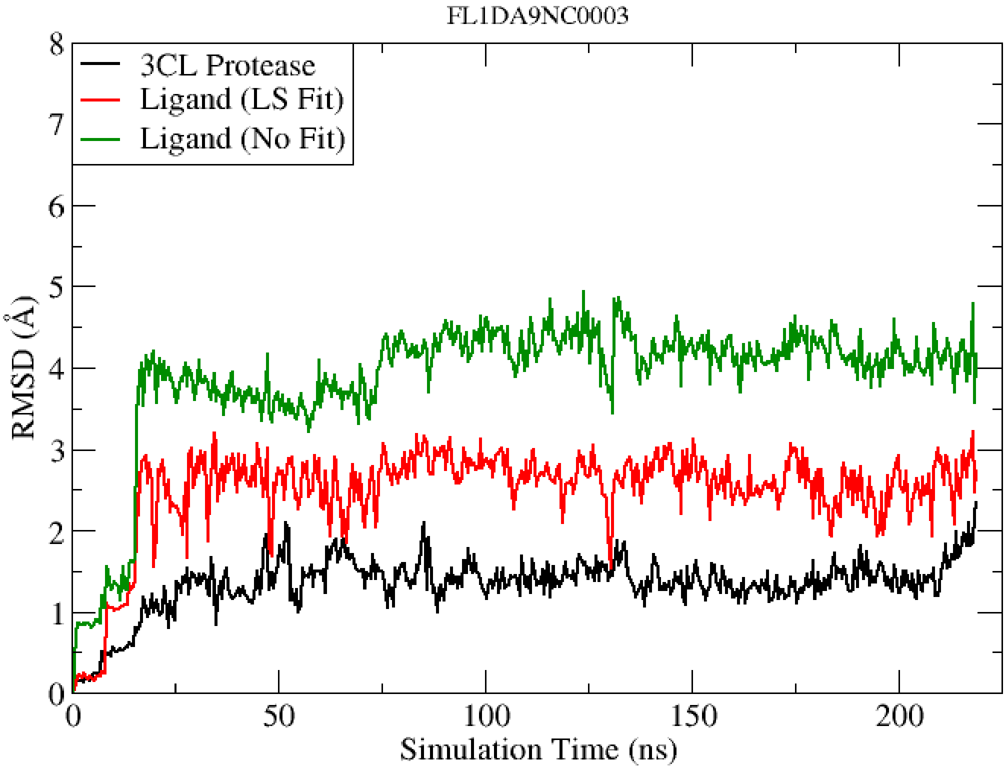

Figure S1. RMSD of the 3CL-pro protein and FL1DA9NC003 ligand with or without least square from the binding simulation for 218 ns. LS, least square fit. RMSD, Root Mean Square Deviation.

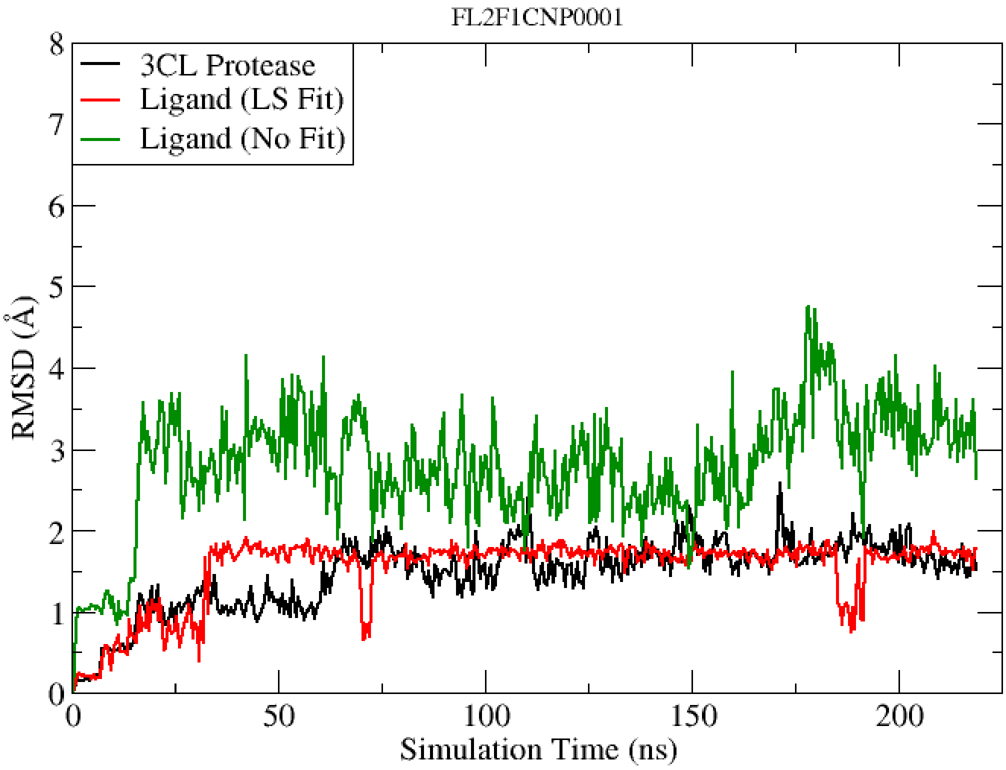

Figure S2. RMSD of the 3CL-pro protein and FL2F1CNP0001 ligand with or without least square from the binding simulation for 218 ns. LS, least square fit. RMSD, Root Mean Square Deviation.

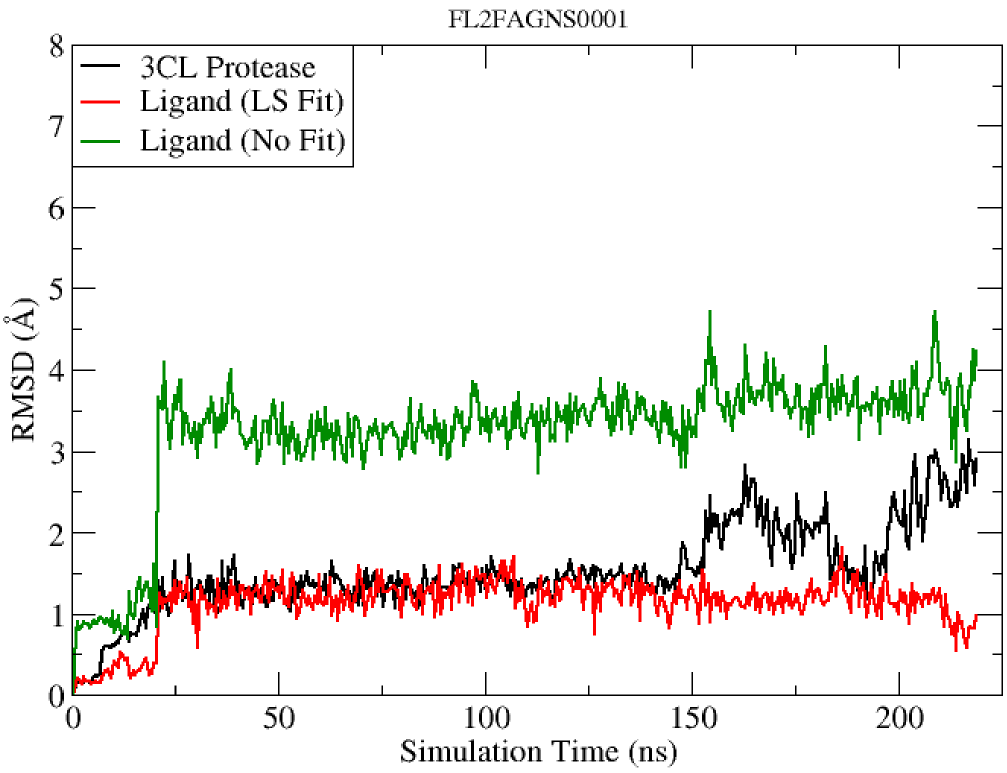

Figure S3. RMSD of the 3CL-pro protein and FL2FAGNS0001 ligand with or without least square from the binding simulation for 218 ns. LS, least square fit. RMSD, Root Mean Square Deviation.

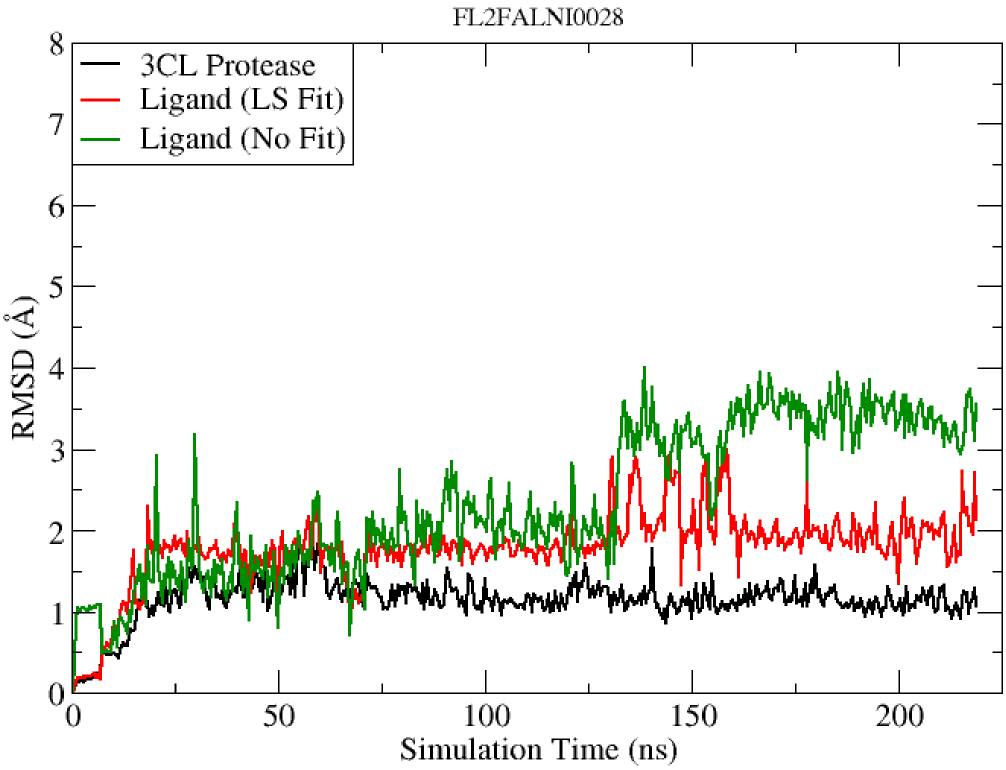

Figure S4. RMSD of the 3CL-pro protein and FL2FALNI0028 ligand with or without least square from the binding simulation for 218 ns. LS, least square fit. RMSD, Root Mean Square Deviation.

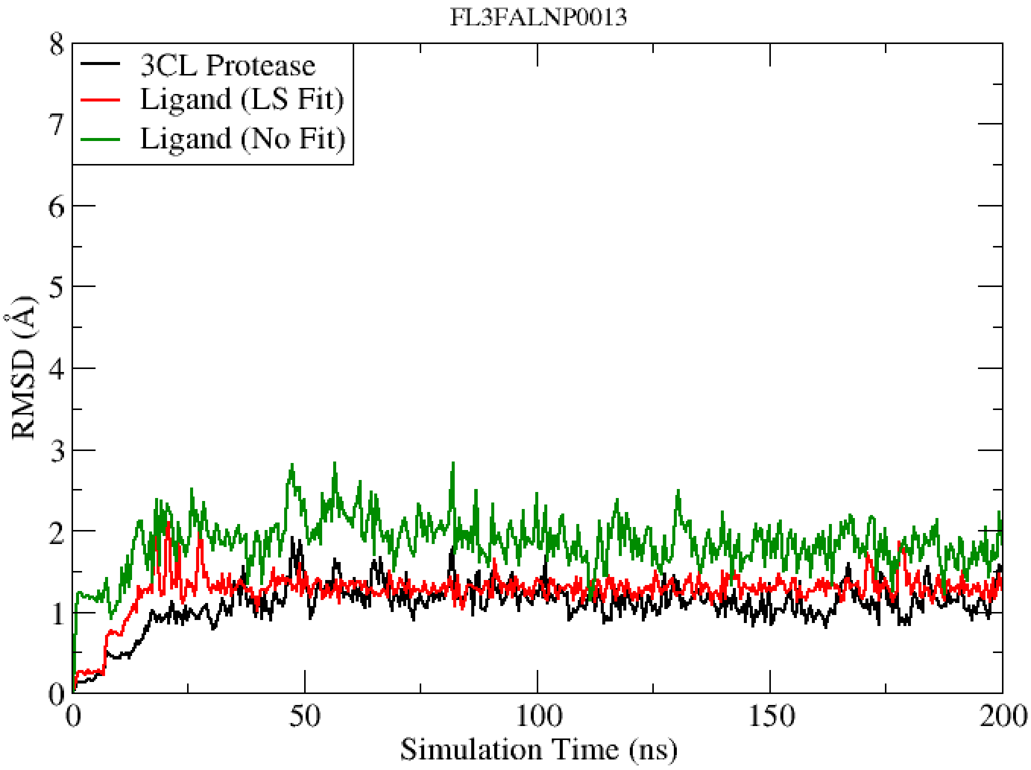

Figure S5. RMSD of the 3CL-pro protein and FL3FALNP0013 ligand with or without least square from the binding simulation for 218 ns. LS, least square fit. RMSD, Root Mean Square Deviation.

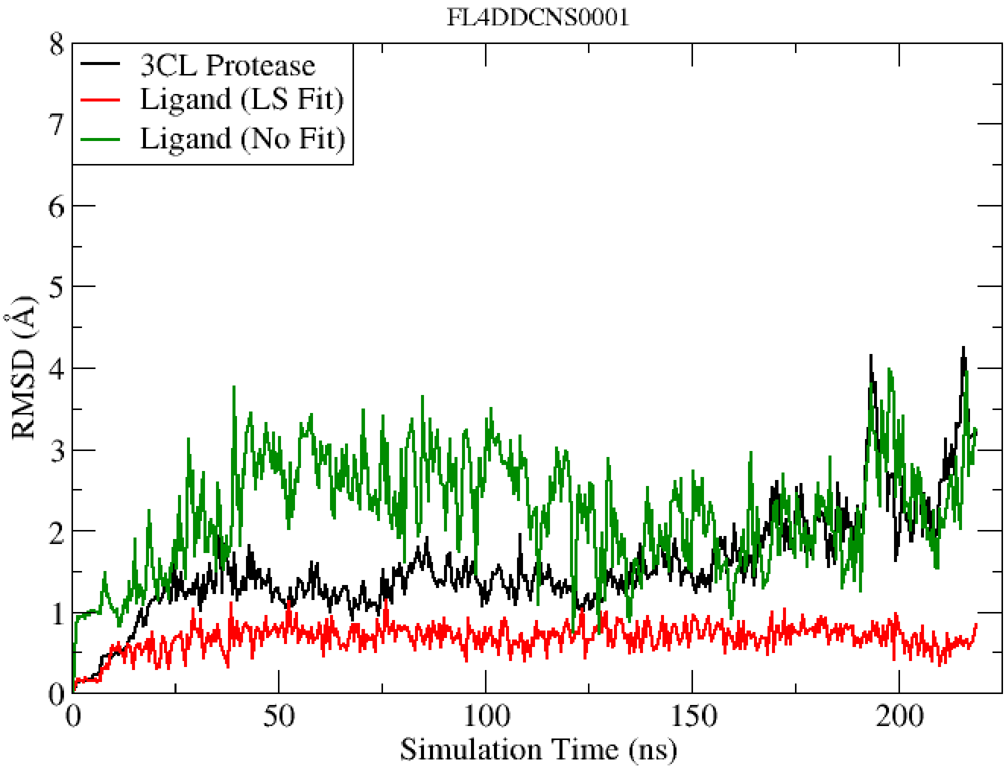

Figure S6. RMSD of the 3CL-pro protein and FL4DDCNS0001 ligand with or without least square from the binding simulation for 218 ns. LS, least square fit. RMSD, Root Mean Square Deviation.

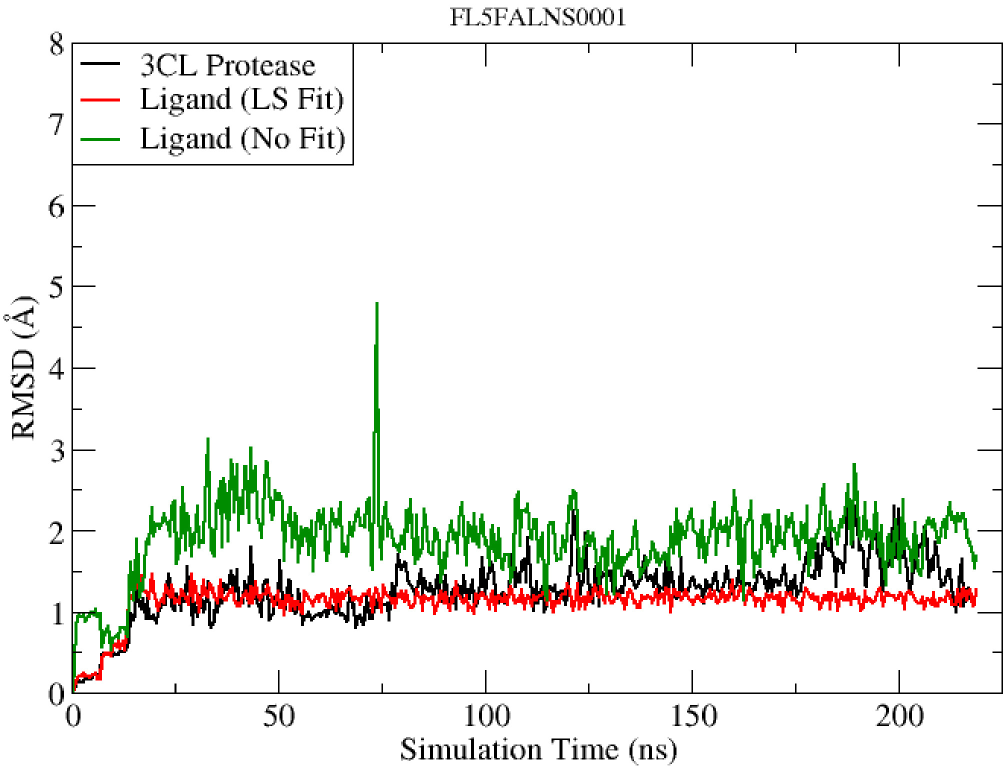

Figure S7. RMSD of the 3CL-pro protein and FL5FALNS0001 ligand with or without least square from the binding simulation for 218 ns. LS, least square fit. RMSD, Root Mean Square Deviation.

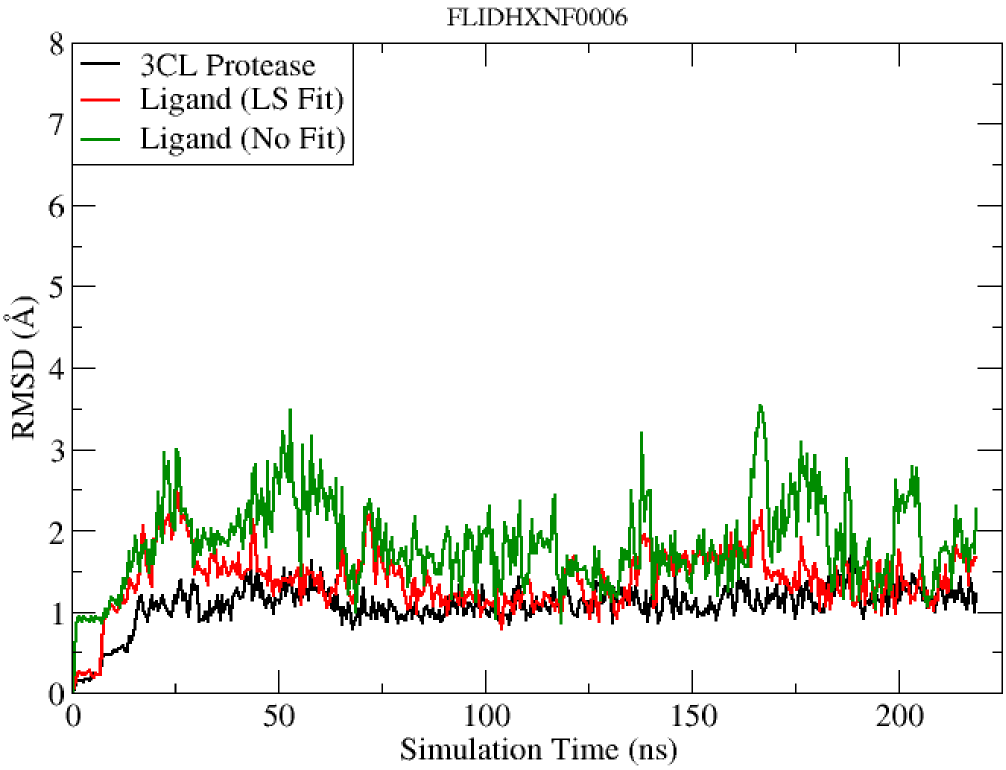

Figure S8. RMSD of the 3CL-pro protein and FLIDHXNF0006 ligand with or without least square from the binding simulation for 218 ns. LS, least square fit. RMSD, Root Mean Square Deviation.

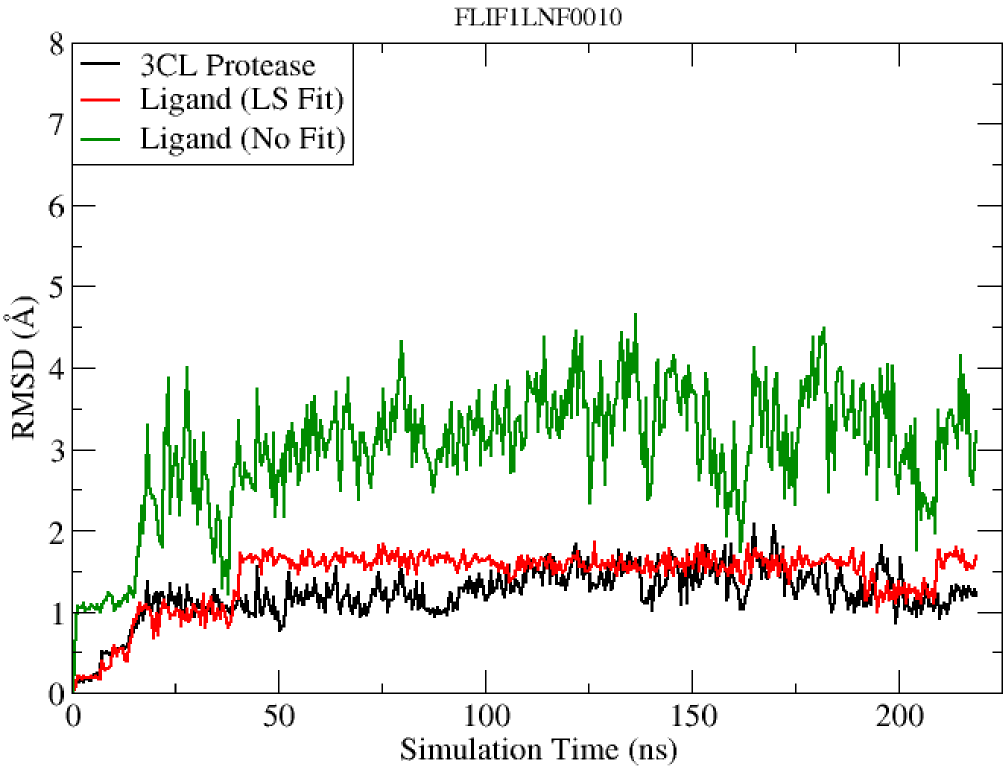

Figure S9. RMSD of the 3CL-pro protein and FLIF1LNF0010 ligand with or without least square from the binding simulation for 218 ns. LS, least square fit. RMSD, Root Mean Square Deviation.
