# Supplementary material for: In Silico Screening of Natural Flavonoids against 3-Chymotrypsin-like Protease of SARS-CoV-2 Using Machine Learning and Molecular Modeling
Source: Molecules. 2023 Dec 10;28(24):8034. doi: 10.3390/molecules28248034 (PMC10745665; doi:10.3390/molecules28248034)
Supplement: Supplementary file 1 [file molecules-28-08034-s001.zip › Tables S5 and S6.pdf]

Table S5. Performance metrics of 20-folds cross-validation results and the assigned training subset as well as ML algorithms for each regression model.

Model	Subset	ML Algorithms	RMSE	CORR2	MAE	# Compounds
Reg1	Set A1	SVM Fine Gaussian	1.549	0.110	1.400	193
Reg2	Set A2	Bagged Trees	1.413	0.250	1.191	193
Reg3	Set A3	Rational Quadratic GPR	1.455	0.200	1.278	193
Reg4	Set A4	Rational Quadratic GPR	1.416	0.240	1.221	193
Reg5	Set A5	Exponential GPR	1.500	0.150	1.348	193
Reg6	Set A6	Exponential GPR	1.508	0.140	1.349	193
Reg7	Set A7	Exponential GPR	1.461	0.190	1.303	193
Reg8	Set A8	Exponential GPR	1.443	0.210	1.280	193
Reg9	Set A9	Rational Quadratic GPR	1.502	0.150	1.346	193
Reg10	Set A10	Exponential GPR	1.482	0.170	1.317	193

RMSE, root mean square error. MAE, mean absolute error. **CORR2**, squared correlation coefficient.

Table S6. Performance metrics of 20-folds cross-validation results and the training set as well as ML algorithm for each classifier.

Model	Subset	ML Algorithms	ACC %	FPR %	AUC	# Compounds
Cla1	Set B1	Bagged Trees	63.29	22.3	0.65	662
Cla2	Set B2	K-Nearest Neighbors	60.39	18.0	0.65	669
Cla3	Set B3	K-Nearest Neighbors	60.94	16.2	0.68	658
Cla4	Set B4	Bagged Trees	62.18	20.1	0.68	669
Cla5	Set B5	K-Nearest Neighbors	60.00	18.5	0.66	655
Cla6	Set B6	K-Nearest Neighbors	60.55	19.5	0.65	659
Cla7	Set B7	Bagged Trees	60.54	21.5	0.66	669
Cla8	Set B8	Bagged Trees	62.80	19.3	0.67	672
Cla9	Set B9	Bagged Trees	60.77	16.9	0.67	673
Cla10	Set B10	Bagged Trees	60.59	17.7	0.67	675
Cla11	Set B11	K-Nearest Neighbors	60.27	18.1	0.65	672
Cla12	Set B12	K-Nearest Neighbors	60.42	15.8	0.65	672
Cla13	Set B13	K-Nearest Neighbors	60.39	13.0	0.67	669
Cla14	Set B14	K-Nearest Neighbors	62.61	15.2	0.69	658
Cla15	Set B15	Bagged Trees	60.36	17.0	0.64	666
Cla16	Set B16	Bagged Trees	60.66	15.8	0.67	666
Cla17	Set B17	Bagged Trees	60.27	21.7	0.67	662
Cla18	Set B18	Bagged Trees	61.14	20.1	0.65	669
Cla19	Set B19	Bagged Trees	61.85	21.3	0.66	658
Cla20	Set B20	Bagged Trees	62.67	15.9	0.68	675
Cla21	Set B5	Fine Gaussian SVM	61.8	6.5	0.68	655
Cla22	Set B11	Fine Gaussian SVM	61.0	4.7	0.68	672
Cla23	Set B2	Fine Gaussian SVM	61.4	4.1	0.67	669
Cla24	Set B9	Fine Gaussian SVM	63.3	2.9	0.65	673
Cla25	Set B16	Fine Gaussian SVM	62.0	4.2	0.66	666

ACC, accuracy. FPR, false positive rate. AUC, area under curves of receiver operating characteristics.
